# The Effect of Quasi-Spherical Gold Nanoparticles on Two-Photon Induced Reactive Oxygen Species for Cell Damage

**DOI:** 10.3390/nano11051180

**Published:** 2021-04-30

**Authors:** Jiunn-Woei Liaw, Chia-Yu Kuo, Shiao-Wen Tsai

**Affiliations:** 1 Department of Mechanical Engineering, Chang Gung University, Taoyuan 333323, Taiwan; markliaw@mail.cgu.edu.tw; 2 Department of Mechanical Engineering, Ming Chi University of Technology, New Taipei City 243303, Taiwan; 3 Medical Physics Research Center, Institute for Radiological Research, Chang Gung University and Chang Gung Memorial Hospital, Taoyuan 333323, Taiwan; 4 Proton and Radiation Therapy Center, Linkou Chang Gung Memorial Hospital, Taoyuan 333423, Taiwan; 5 Graduate Institute of Biomedical Engineering, Chang Gung University, Taoyuan 333323, Taiwan; kmhk181@yahoo.com.tw; 6 Department of Periodontics, Chang Gung Memorial Hospital, Taipei 105406, Taiwan

**Keywords:** reactive oxygen species, gold nanoparticles, two-photon, hot electrons, cell damage, laser scanning confocal microscopy, cytoskeleton disruption, apoptosis, blebbing, NAC

## Abstract

The performance of quasi-spherical gold nanoparticles (GNPs) on the generation of reactive oxygen species (ROS) to cause cell damage, as irradiated by a two-photon laser, is studied. In this mechanism, hot electrons are generated from GNPs as irradiated by the two-photon laser, reacting with the molecules in the medium to produce ROS. We used laser scanning confocal microscopy with a low-fluence femtosecond Ti:Sapphire laser of 800 nm to observe the generated ROS in A431 cells, which were incubated with GNPs in advance. Subsequently, the cell morphology, cytoskeleton, and viability were investigated. In comparison with the control (no GNPs), the expression of ROS in these GNP-treated cells was enhanced after irradiation by the two-photon laser. Additionally, the disruption of cytoskeletons and the follow-up apoptosis of these GNP-treated cells are significantly increased as the number of laser shots increases. Moreover, we used N-acetyl-L-cysteine (NAC), an antioxidant, to inhibit the formation of ROS, to clarify whether the cytoskeletal disruption is caused by ROS rather than photothermal effects. Our results show that after two-photon irradiation, the ROS expression in these cells treated with GNPs plus NAC was significantly reduced. In addition, the cytoskeletal damage of these cells treated with GNPs and NAC was less than that of those treated with GNPs but without NAC; their cell viability after three days was almost the same with the control. These results illustrate that the induced ROS from the two-photon excited GNPs is the main cause of cell damage. The study may pave a way for the use of GNPs as a photosensitized therapeutic agent for two-photon photodynamic therapy on tumor treatment.

## 1. Introduction

Gold nanoparticles (GNPs) attract attention and are widely used in biomedical applications due to their specific photothermal properties, good biocompatibility, and simple surface modification [1,2,3]. The photothermal effect is due to the surface plasmon resonance (SPR) of GNPs, which is the collective motion of free electrons interacting with photons; the energy of photons in the region of visible light to near infrared (NIR) can be converted into heat. In recent decades, numerous of methods with different surfactants and reduction reagents were proposed to fabricate GNPs of various shapes and sizes for tuning the SPR property. Several biomedical applications of photothermal effect of GNPs were developed, e.g., hyperthermia therapy. Through the endocytosis of cells, the uptake GNPs can be accumulated in vesicles of cytoplasm. Under continuous wave (CW) laser irradiation, the photothermal effect of GNPs occurs at a localized area in the cytoplasm to induce damage to organelles. For example, blebbing of the membranes of cells treated with GNPs was observed [4]. If a high-power CW laser is used to irradiate cells treated with GNPs, the generated heat damages proteins and other indispensable enzyme structures in the vicinity of GNPs, which leads to cell necrosis [5,6]. In addition, the apoptosis of these GNP-treated cells irradiated by a nanosecond-pulsed laser was also reported [7]. This is because that the transient heat around GNPs induces nanobubbles to disrupt the cytoskeletons, leading to cell lysis [7]. On the other hand, a mild irradiation of GNP-treated cells with a low-power laser may promote cell differentiation [8]. Hence, the cellular fate can be different depending on the type of laser, fluence and irradiation time. 

Recently, using the two-photon effect of femtosecond NIR lasers to generate reactive oxygen species (ROS) in media or tissue has drawn a lot of attention [9,10]. Two-photon excitation by a femtosecond laser with ultra-short pulses to generate hot electrons is a nonlinear effect. Normally, the efficiency of a femtosecond NIR laser (e.g., 800 nm) is higher than that of a CW laser (e.g., 405 nm). The ROS, such as hydroxyl radicals (OH·) and superoxide anion O_2_^−^, is essential in biological systems to maintain a stable redox state. However, excessive ROS could damage cellular organelles (e.g., mitochondria and cytoskeletons), and induce blebbing and even apoptosis and necrosis [11,12,13]. Moreover, the ROS induced by two-photon excitation can be enhanced via the SPR of GNPs [14,15,16,17,18,19,20,21,22,23]. The mechanism is that hot electrons can be generated from GNPs by irradiation with a two-photon laser beam, which then react with the molecules in the medium to produce ROS [24]. Numerous studies have demonstrated that GNPs or gold nanorods, similar to photosensitizers, can enhance apoptosis of cancer cells in photodynamic therapy [25,26,27]. Since NIR light is in the transparency window of biological tissue, an NIR laser is preferred for use in non-invasive photodynamic therapy. Compared to traditional photosensitizers, GNPs have several advantages, such as high photostability, good biocompatibility, low cytotoxicity, high-efficiency in hot-electron generation, and the extra photothermal effect. However, the disadvantage is that they are non-biodegradable. This means that if the accumulated dose of GNPs is over an acceptable value, the cytotoxicity is induced to varying degrees. The femtosecond NIR laser is a promising two-photon source for the treatment of malignant tissues due to the deep tissue penetration and high spatial resolution [28]. Recently, several studies combining two-photon excitation with GNPs in photodynamic therapy have shown significant progress [14,15,16,17,18,19,20,21,22,23]. For instance, Han et al. [29] reported that dihydrolipoic acid-coated gold nanoclusters could be new photosensitizers with excellent two-photon absorption and strong ROS generation ability. Gao et al. [30,31] concluded that gold nanocage-PEG mediated under two-photon irradiation can produce more ROS than under CW laser excitation. However, several studies have indicated that low concentrations of ROS can promote cell proliferation and differentiation [32,33]. Thus, it is crucial to control the dose of induced ROS in cells for different purposes. To our knowledge, few studies investigated the detailed biomedical effects of the combination of low-power two-photon excitation with GNPs in relation to ROS production [34,35]. Therefore, the present work focuses on the study of GNPs excited by two photons in the generation of ROS, and the resulting influence on cell damage (e.g., cytoskeletal disruption) and viability.

The major purpose of this paper is to study the generated ROS from GNPs on cell damage, so that any other factors inducing cytotoxicity need to be avoided. However, the surfactant is necessary for the synthesis of the gold nanorods and nanotriangles. Due to the cytotoxicity of surfactants, we used the surfactant-free- and quasi-spherical GNPs rather than gold nanorods or nanotriangles, even though they have a higher two-photon effect. In addition, the uptake of quasi-spherical GNPs of 55 nm in size by cells is better than the other shaped GNPs (e.g., nanostars). Additionally, a femtosecond NIR laser of was used in our research to induce hot electrons for generating ROS by the nonlinear two-photon effect, inducing less heat. Recently, the photodynamic therapy has been applied in the treatment of breast cancer, particularly on multidrug-resistant cancer cells [36,37,38]. Therefore, our aim is to study the feasibility of using GNPs as photosensitizer for the photodynamic therapy with two-photon lasers in the treatment of breast cancer. Since A431 cells (a human epidermoid carcinoma cell line) form a kind of breast cancer cell line, we chose this cell line for our study.

## 2. Materials and Methods

All chemicals used in the present study were purchased from Sigma-Aldrich Chemical Company (St. Louis, MO, USA) unless otherwise stated. The experiments were conducted under a laser scanning confocal microscope (LSCM) (Zeiss LSM 510 META, Germany) with a femtosecond Ti:Sapphire laser (Mai Tai with a repetition rate of 116 MHz, Spectra Physics) of 800-nm wavelength at 20% power of 1.5 W for irradiation. A 100× objective (NA: 1.4) was used for scanning, where the spot size was about 4 μm^2^. The two-photon irradiation was a 2D point scan in a region of 90 µm × 90 µm with 1024 × 1024 points, where the pixel time of laser irradiation was 1.6 µs at each point. When the output power through all optical lenses was 70%, the average fluence was 8 J/cm^2^ at each point.

### 2.1. Preparation and Characterization of GNPs

The method of synthesizing quasi-spherical GNPs by reducing a gold salt by citrate was used based on a method in a previous study [2]. Briefly, 100 µL of 0.5 M HAuCl_4_ and 120 µL of 1% sodium citrate were added to 200 mL of deionized water and boiled for 15 min. The average size of the GNPs synthesized in the study was 55.08 ± 3.21 nm, which was determined by randomly selecting 100 particles in transmission electron microscopy (TEM) images, and the absorption spectrum showed a major SPR adsorption peak at 536 nm (Supplement Figure S1). The concentrations of GNP-colloids were measured via an inductively coupled plasma atomic emission spectroscopy (ICP-OES).

### 2.2. Cell Culture

In this study, A431 cells (a human epidermoid carcinoma cell line, BCRC 60161) were used to investigate the biological effect of the induced ROS from two-photon excited GNPs. The culture medium of A431 cells was Dulbecco’s Modified Eagle Medium (DMEM) supplemented with 10% fetal bovine serum and 1% antimicrobial agent. These cells were maintained at 37 °C in a humidified atmosphere with 5% CO_2_, and we refreshed the medium once every 3 days. 

### 2.3. Optical Imaging of Cells

Cytoskeleton- and viability staining were used to investigate the effect of ROS induced from GNPs interacting with a two-photon laser on cells. Hoechst 33342 and Texas Red-X phalloidin (Thermo Fisher Scientific, Waltham, MA, USA) were used to stain the nucleus and the F-actin cytoskeleton, respectively, for the image of LSCM. In addition, cell viability was determined by a fluorescent dye kit (LIVE/DEAD^®^ Viability/Cytotoxicity Kit L-3224; Molecular Probes, Carlsbad, CA, USA) according to the manufacturer’s protocol. In brief, after laser irradiation, 200 µL of dye reagent containing 1 µM calcein AM and 2 µM ethidium homodimer-1 were added in the cell medium at 37 °C for 20 min. After being irradiated by a femtosecond laser with 20% power, the cells were washed three times with phosphate-buffered saline (PBS), then fixed in a 4% paraformaldehyde solution for 15 min at room temperature, and finally treated with 0.1% Triton X-100 for 5 min. The samples were blocked with 1% bovine serum albumin (BSA) in PBS for another 30 min to reduce nonspecific background staining. After washing out the BSA solution, the samples were incubated with Texas Red-X phalloidin for 20 min. The samples were then incubated with Hoechst 33342 solution for 5 min to stain the DNA in the nuclei of these cells. The images of the samples were then observed under an LSCM (Zeiss LSM 510 META) after being washed with PBS several times. 

### 2.4. Detection of ROS Generation

A DCFDA cellular ROS assay kit (Abcam, Cambridge, MA, USA) was used to label ROS. In brief, after laser irradiation, the samples were washed three times with PBS, and then the DCFDA solution was added to the cell medium under low light conditions and incubated for 20 min. The samples were then incubated with Hoechst 33342 solution for 5 min, followed by washing with PBS. The samples were then observed under an LSCM (Zeiss LSM 510 META); the fluorescence intensity of ROS kit was semi-quantified by Image J software to represent the amount of generated ROS.

To further clarify the relationship between biological effect and the dose of ROS generated from the two-photon excited GNPs, N-acetyl-L-cysteine (NAC), which is an inhibitor of ROS [39,40], was added to the cell medium for 1 h before laser irradiation. 

### 2.5. Statistical Analyses

All experiments were conducted in independent triplicates and, each time, three samples were tested in parallel. Statistical analyses were performed using the SPSS v.17 software package. The results of statistics were expressed as the mean ± SDs. A nonparametric test was used to analyze the differences between different groups; *p* < 0.05 was considered to be statistically significant.

## 3. Results and Discussion

Numerous factors, such as size, surfactant, surface charge and concentration of GNPs, cell type, and GNP treatment time, affected the cytotoxicity. Therefore, we characterized the GNPs and determined the optimal culture conditions before conducting laser irradiation. Because cells generate ROS after the uptake of GNPs, the concentration of GNP colloid and incubation time must be well controlled to prevent the cell apoptosis induced by high ROS levels. Based on our few preliminary studies (Supplementary Figure S2), cells incubated with 35-ppm GNPs for 3 h produced an optimal condition to investigate the responses of cells to the generated ROS from two-photon-excited GNPs.

**Figure 1:**
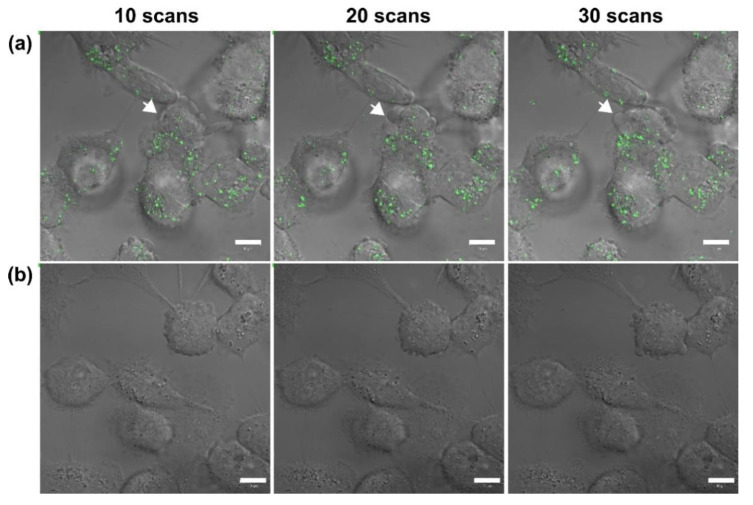
Bright-field images of (**a**) GNP-treated A431 cells irradiated by two-photon laser with 10, 20, and 30 scans, and (**b**) the control group (without GNPs). The green spots are the scattering expression from GNPs accumulated in vesicles through organelle fusion. Scale bar: 10 µm. The images of GNP-treated cells show the blebbing at cellular membranes.

**Figure 2:**
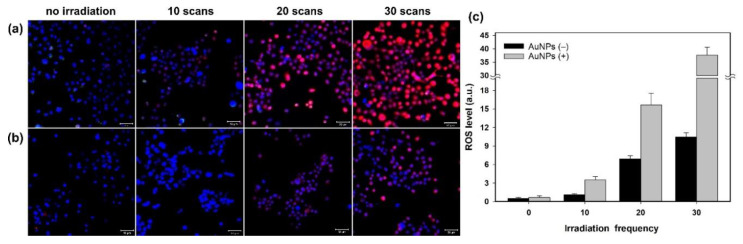
Fluorescence images of LSCM for ROS expression. Images of (**a**) A431 cells treated with GNPs irradiated by a two-photon laser with 10, 20, and 30 scans, and (**b**) the control group (cells not treated with GNPs). (**c**) Quantitative comparison of generated ROS of (**a**,**b**). Scale bar: 50 µm. The kit for detecting ROS is DCFDA (red fluorescence), and the kit for staining nuclei is Hoechst 33342 (blue fluorescence).

**Figure 3:**
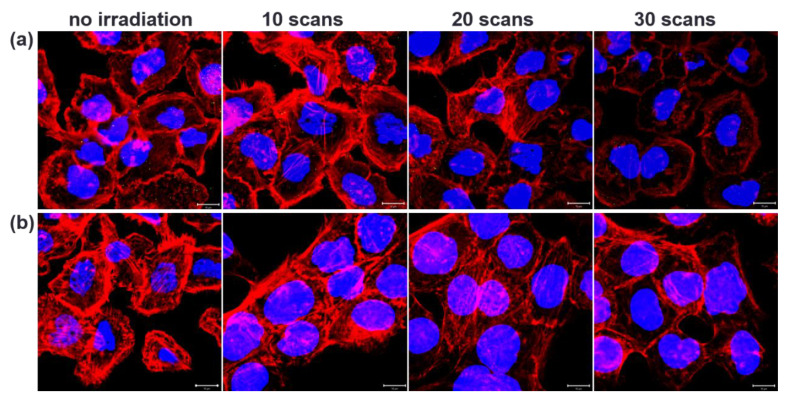
Fluorescence images of LSCM for cellular cytoskeleton. Images of (**a**) A431 cells treated with GNPs irradiated by two-photon laser with 10, 20, and 30 scans, and (**b**) the control group (cells not treated with GNPs). Texas Red-X phalloidin was used to label cytoskeletal F-actin (red), and Hoechst 33342 was used to stain nuclei (blue). Scale bar: 10 µm.

In this study, we used a two-photon laser of 800 nm with 20% power for irradiation; the average fluence at each laser spot was 8 J/cm^2^. As shown in Figure 1a, we observed the membrane blebbing of these GNP-treated cells after two-photon irradiation of 10, 20, and 30 scans from the bright-field images. The green spots are the scattering expression from GNPs accumulated in vesicles through organelle fusion [3]. In fact, the bare GNPs are dispersed in the medium (DMEM). However, the absorption spectrum of GNPs in the medium (DMEM) becomes broadened in comparison with that in water, and the SPR peak is red-shifted to 565 nm (Supplementary Figure S1c). This change in optical property could be attributed to the degree of aggregation of GNPs in the medium (DMEM) and the higher refractive index of the medium relative to water. Although the GNPs are internalized individually by cell, these internalized GNPs are eventually aggregated in vesicles through the organelle fusion after the cellular endocytosis. This natural aggregation of GNPs in cellular vesicles makes the surface plasmon resonance red-shifted and broadband [41] Therefore, the two-photon effect of femtosecond NIR laser on these clustered GNPs with a small gap between GNPs in vesicles is enhanced. In contrast, there is no blebbing in the morphology of the A431 cells without GNPs after irradiation of 10, 20 and 30 scans (see Figure 1b). We deduced that this phenomenon of blebbing was due to the induced ROS from the two-photon excited GNPs causing oxidative stress and leading to damage to the cytoskeleton [29]. To confirm this hypothesis, we also detected the amount of ROS generated at different numbers of two-photon irradiation scans. Figure 2 shows that the ROS amount increases with the number of irradiations for both A431 cells with and without GNP treatment. Moreover, we found that the expression of ROS for these GNP-treated cells is more significant than the untreated cells at 20 and 30 scans by comparing Figure 2a with Figure 2b. Using Image J to quantify ROS generation (Figure 2c), we also found that the ROS amount in GNP-treated cells increased 2.3-fold and 3.6-fold after irradiation of 20 and 30 scans compared to that of the control sample, respectively. Our results indicated that GNPs can enhance the generation of ROS as irradiated by two-photon laser; the amount of ROS increases with the increase in scan number for two-photon irradiation. ROS are essential in biological systems; the specific-level maintenance of ROS can promote cell proliferation and differentiation. However, if the concentration of ROS in a cell is too high, it may induce oxidative damage of DNA, lipids, and proteins [42]. For example, the continuous F-actin and microfilaments may be broken into several segments by ROS, under high oxidation pressure. Therefore, the cytoskeletal structure and cell cortex can be damaged by excessive ROS. This damage may cause cellular blebbing, and then increase membrane permeability to cause an unbalanced ionic gradient in cells, finally leading to apoptosis or even necrosis [43,44]. Moreover, we used LSCM image to study the integrity of the cytoskeletons. The images of Figure 3a further indicated that the cytoskeletons of these GNP-treated cells were significantly broken after the two-photon irradiation. The disruption of the cytoskeletons was more serious with the increasing scan numbers of irradiation. The results of cytoskeletal damage were consistent with the amounts of generated ROS, which broke the F-action filaments of the cytoskeletons and cortexes into shorter segments to cause membrane blebbing (see Figure 1a and Figure 3a). In contrast, the morphology of the untreated cells’ cytoskeletons after 30 scans of irradiation was relatively continuous, as shown in Figure 3b. 

**Figure 4:**
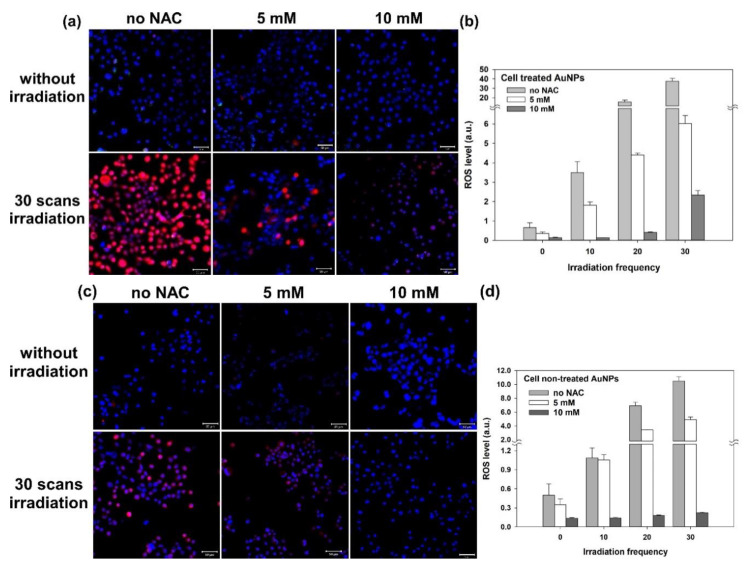
Fluorescence images of LSCM for A431 cells irradiated by two-photon laser showing the inhibiting effect of NAC on ROS production. (**a**) ROS-expression fluorescence (red) images of cells treated with GNPs and (**b**) the corresponding quantitative ROS generation for 0, 10, 20, and 30 scans of two-photon irradiation. (**c**) ROS-expression fluorescence (red) images of the control group (cells not treated with GNPs) and (**d**) the corresponding quantitative ROS generation. Hoechst 33342 was used to stain nuclei (blue), and red fluorescence was from the DCFDA cellular ROS assay kit. Scale bar: 50 µm.

**Figure 5:**
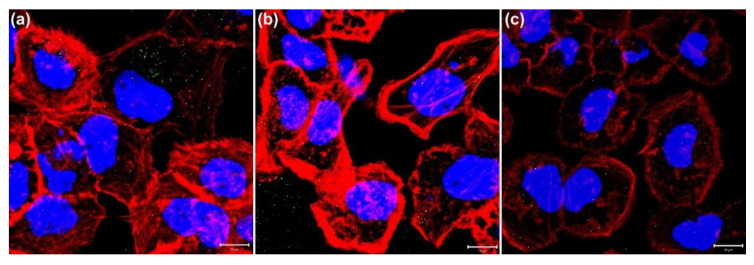
Fluorescence images of cellular cytoskeleton (LSCM) to show the inhibiting effect of NAC on ROS. Images of GNP-treated A431 cells treated with NAC of (**a**) 5 mM and (**b**) 10 mM irradiated by two-photon laser with 30 scans. (**c**) Image of GNP-treated A431 cells without NAC. Texas Red-X phalloidin was used to label cytoskeletal F-actin (red), and Hoechst 33342 was used to stain nuclei (blue). Scale bar: 10 µm.

**Figure 6:**
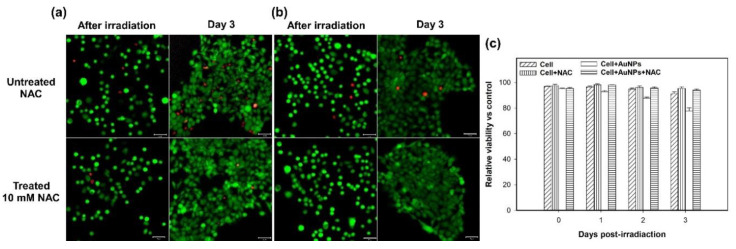
Fluorescence microscopy images of live (green)/dead (red) staining of A431 cells irradiated by two-photon laser after 3 days. Images of (**a**) GNP-treated cells with or without 10-mM NAC, and (**b**) the control group (not treated with GNPs) with or without 10-mM NAC. (**c**) The cell viability versus days of post-irradiation (0, 1, 2, 3).

Furthermore, we clarified that the above-mentioned cell damage (disruption of the cytoskeleton) was mainly caused by ROS rather than the photothermal effect of GNPs. Thus, we added NAC (5 mM or 10 mM) in the medium to inhibit the ROS induced from the two-photon excited GNPs. NAC is an antioxidant that acts as a scavenger of ROS. The results, shown in Figure 4, illustrate that the expression of ROS is significantly reduced in these NAC-treated cells with and without GNPs after the two-photon irradiation. As we raised the concentration of NAC (e.g., 10 mM), the inhibiting effect on ROS increased. For the GNP-treated cells irradiated by a two-photon laser for 30 scans, the expression of the induced ROS was clearly inhibited by NAC (Figure 4a–d). In addition, these cells’ membrane integrity was good, with less blebbing. As shown in Figure 5a,b, there was less cytoskeletal disruption in the cells treated by GNPs and NAC (5 mM and 10 mM) after two-photon laser irradiation, compared to those cells only treated by GNPs but without NAC (Figure 5c). The more the NAC, the less the cytoskeletal disruption. After three days, the cellular viability of these NAC-treated cells irradiated by a two-photon laser was almost the same as the control (without the treatment of GNPs and NAC), as shown in Figure 6a,b. In contrast, the cell viabilities of those GNP-treated cells without NAC were the lowest compared to the other cells after two-photon irradiation for 1, 2, and 3 days (Figure 6c). Based on these results, we concluded that the generated ROS from two-photon excited GNPs was the major cause of cell damage and apoptosis, rather than the photothermal effect. 

## 4. Conclusions

In this paper, we studied the enhancement effect of quasi-spherical GNPs, irradiated by a two-photon laser, on the generation of ROS. The excessive ROS can be used to cause damage to cellular organelles (e.g., mitochondria and cytoskeleton), and even induce apoptosis. The mechanism is that hot electrons generated from GNPs, irradiated by the two-photon laser, react with the molecules in the medium to produce ROS. Through the organelle fusion, these internalized GNPs are aggregated in vesicles of cytoplasm to make the SPR redshifted and broadband. We used LSCM combined with a femtosecond laser to induce ROS and observed the expression of ROS in A431 cells, which were incubated with GNPs in advance. Subsequently, the cell morphology, viability, and apoptosis were investigated. In particular, the cytoskeletal disruption of these cells caused by the induced ROS was investigated. In comparison with the control (cells without GNPs), we found that the expression of ROS in these GNP-treated cells was enhanced after the two-photon irradiation. Additionally, the disruption of cytoskeletons and the apoptosis of these GNP-treated cells was significantly increased, compared to the untreated cells. As to the cellular morphology, we observed blebbing at the cellular membranes and at the giant plasma membrane vesicle. Blebbing indicates the disruption of cytoskeletons. As we increased the number of two-photon scans, the expression of ROS was increased; the damage to cytoskeletons and apoptosis was also positively correlated to the number of laser shots. Moreover, we used NAC, an antioxidant, to inhibit the formation of ROS. Our results show that after the two-photon irradiation, the expression of ROS in cells treated by GNPs and NAC was significantly inhibited. In addition, cytoskeletal damage and apoptosis after three days were significantly reduced. When we increased the dose of NAC, the cytoskeletal damage and the apoptosis rate of these cells were further reduced. Our results clarify that the cytoskeleton disruption and cellular apoptosis are due to the induced ROS from GNPs, rather than the photothermal effect of GNPs. Recently, two-photon photodynamic therapy has become an important cancer treatment modality [18,29]. Due to the SPR of GNPs, the generated hot electrons can assist the formation of ROS when irradiated by a two-photon laser of low power. This study shows the feasibility of using GNPs as a photosensitized therapeutic agent to enhance the efficacy of two-photon photodynamic therapy for tumor treatment. In future, differently shaped GNPs without surfactant (e.g., protein-coated gold nanorods), for which the efficiency of hot-electron generation is high, may be suitable for biomedical application [45].

## Data Availability

The A431 cell line (a human epidermoid carcinoma cell line, BCRC 60161) was provided by Bioresource Collection and Research Center, Taiwan.

## Supplementary Materials

The following are available online at https://www.mdpi.com/article/10.3390/nano11051180/s1, Figure S1: Characterizations of GNPs. (a) TEM image (×100 K) of GNPs. (b) UV-Vis absorption spectrum of GNPs in water. (c) UV-Vis absorption spectrum of GNPs in medium (DMEM), Figure S2: Amounts of GNPs taken up by A431 cells. (a) A dark-field image showing A431 cells after uptake of GNPs for 3 h. The bright spots represent scattering from GNPs. (b) The dark-field image of control (without GNPs). (c) The total amounts of GNPs which were taken up by A431 cells after treatment with 35-ppm GNPs for 1 and 3 h. GNP concentrations as measured by ICP-OES. (d) The cell viability of GNP-treated cells for 1 and 3 h. Data are presented as the mean ± SD (n = 9), and differences at *p* < 0.05 were considered statistically significant. Scale bar: 10 µm.
