# Supplementary material for: The Effect of Quasi-Spherical Gold Nanoparticles on Two-Photon Induced Reactive Oxygen Species for Cell Damage
Source: Nanomaterials (Basel). 2021 Apr 30;11(5):1180. doi: 10.3390/nano11051180 (PMC8145056; doi:10.3390/nano11051180)
Supplement: Supplementary file 1 [file nanomaterials-11-01180-s001.zip › nanomaterials-1191142-supplementary.pdf]

nanomaterials

Supplementary Materials

The Effect of Quasi-Spherical Gold Nanoparticles on Two-Photon Induced Reactive Oxygen Species for Cell Damage

Jiunn-Woei Liaw [1,2,3,4], Chia-Yu Kuo [5] and Shiao-Wen Tsai [5,6,*]

1 Department of Mechanical Engineering, Chang Gung University, Taoyuan 333323, Taiwan; markliaw@mail.cgu.edu.tw
2 Department of Mechanical Engineering, Ming Chi University of Technology, New Taipei City 243303, Taiwan
3 Medical Physics Research Center, Institute for Radiological Research, Chang Gung University and Chang Gung Memorial Hospital, Taoyuan 333323, Taiwan
4 Proton and Radiation Therapy Center, Linkou Chang Gung Memorial Hospital, Taoyuan 333423, Taiwan
5 Graduate Institute of Biomedical Engineering, Chang Gung University, Taoyuan 333323, Taiwan; kmhk181@yahoo.com.tw
6 Department of Periodontics, Chang Gung Memorial Hospital, Taipei 105406, Taiwan
* Correspondence: swtsai@mail.cgu.edu.tw

The characterizations of GNPs are shown in Figure S1. Figure S1a is a TEM image (x100K) of 55-nm GNPs. Figure S1b shows the UV-Vis absorption spectrum of GNPs, and Figure S1c is the UV-Vis absorption spectrum of GNPs in medium (DMEM). The plasmonic peak of GNPs in water is at 536 nm, while that of GNPs in medium (DMEM) is red-shifted to 565 nm. In addition, the absorption spectrum of GNPs in medium becomes broadened. This change in optical property could be attributed to two factors: the more or less aggregation of GNPs in medium and the relatively higher refractive index of medium.

The dark-field image of A431 cells after uptake of GNPs for 3 hours is shown in Figure S2a, and that of control is in Figure S2b. The bright spots represent scattering from GNPs. The amounts of GNPs taken up by A431 cells after treatment with 35-ppm GNPs for 1 and 3 hours were measured by ICP-OES, as shown in Figure S2c. In addition, Figure S2d shows the cell viability of GNP-treated cells for 1 and 3 hours. The high viability demonstrates that the cytotoxicity of 35-ppm GNPs is much less.

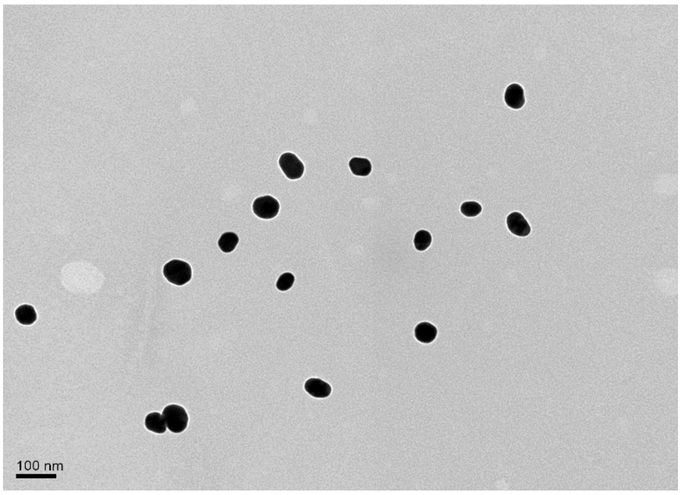

(**a**)

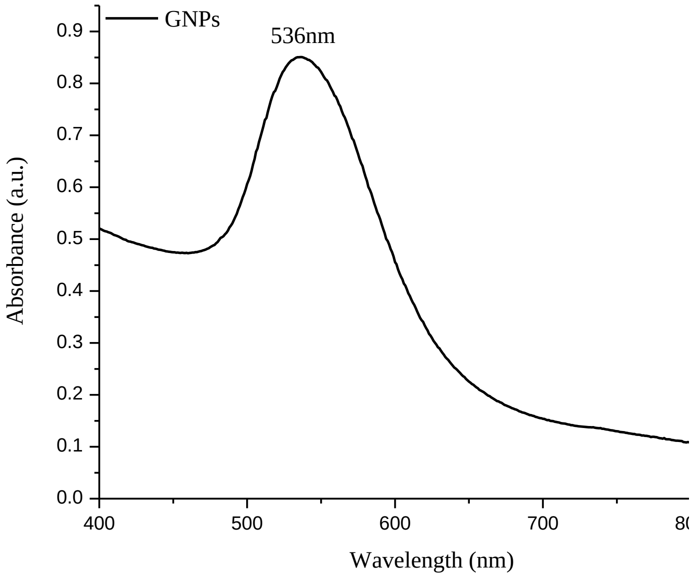

(**b**)

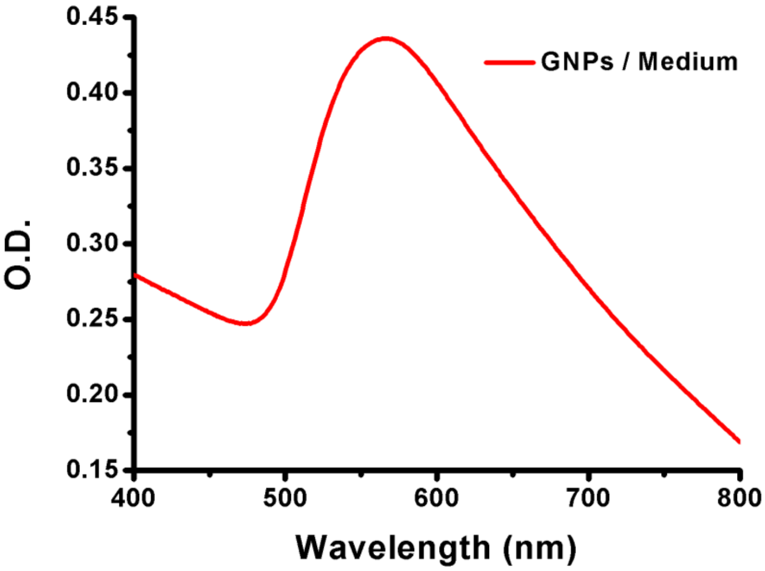

(c)

Figure S1. Characterizations of GNPs. (**a**)TEM image (×100 K) of GNPs. (**b**) UV-Vis absorption spectrum of GNPs in water. (**c**) UV-Vis absorption spectrum of GNPs in medium (DMEM).

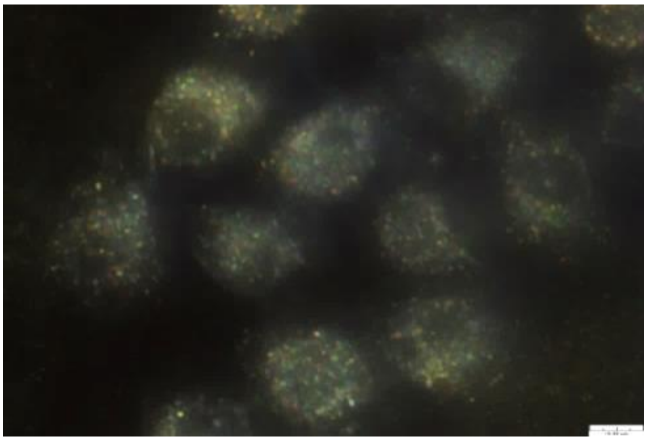

(**a**)

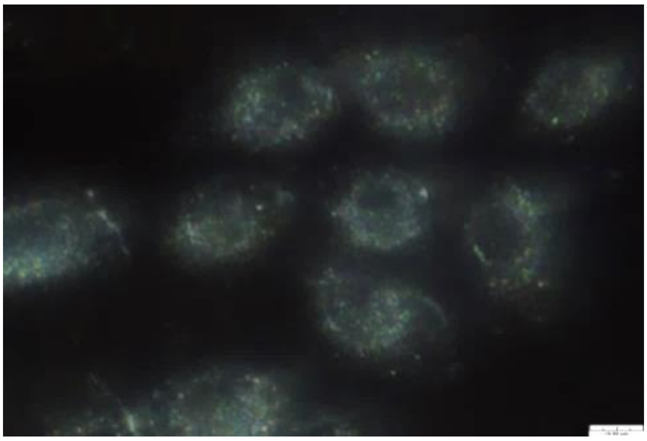

(**b**)

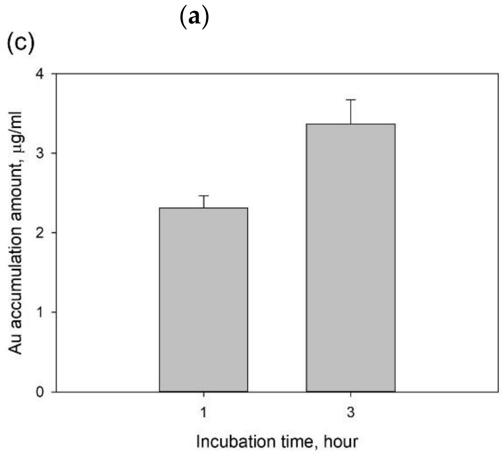

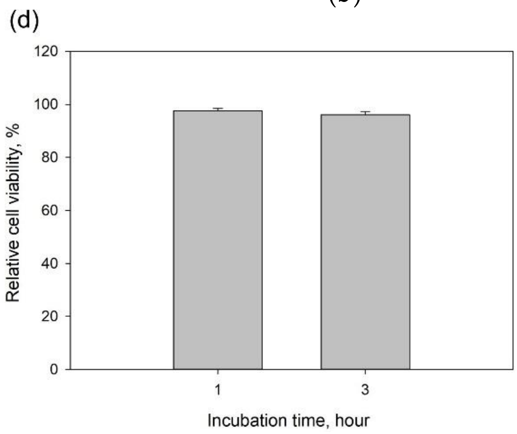

(c)

Figure S2. Amounts of GNPs taken up by A431 cells. (**a**) A dark-field image showing A431 cells after uptake of GNPs for 3 hours. The bright spots represent scattering from GNPs. (**b**) The dark-field image of control (without GNPs). (**c**) The total amounts of GNPs which were taken up by A431 cells after treatment with 35-ppm GNPs for 1 and 3 hours. GNP concentrations as measured by ICP-OES. (**d**) The cell viability of GNP-treated cells for 1 and 3 hours. Data are presented as the mean ± SD (n = 9), and differences at $p < 0.05$ were considered statistically significant. Scale bar: 10 μm.
